# Lignocellulosic Biomass: Understanding Recalcitrance and Predicting Hydrolysis

**DOI:** 10.3389/fchem.2019.00874

**Published:** 2019-12-18

**Authors:** Aya Zoghlami, Gabriel Paës

**Affiliations:** FARE Laboratory, INRAE, University of Reims Champagne-Ardenne, Reims, France

**Keywords:** lignocellulose, recalcitrance, chemical composition, structure, enzymatic hydrolysis

## Abstract

Lignocellulosic biomass (LB) is an abundant and renewable resource from plants mainly composed of polysaccharides (cellulose and hemicelluloses) and an aromatic polymer (lignin). LB has a high potential as an alternative to fossil resources to produce second-generation biofuels and biosourced chemicals and materials without compromising global food security. One of the major limitations to LB valorisation is its recalcitrance to enzymatic hydrolysis caused by the heterogeneous multi-scale structure of plant cell walls. Factors affecting LB recalcitrance are strongly interconnected and difficult to dissociate. They can be divided into structural factors (cellulose specific surface area, cellulose crystallinity, degree of polymerization, pore size and volume) and chemical factors (composition and content in lignin, hemicelluloses, acetyl groups). Goal of this review is to propose an up-to-date survey of the relative impact of chemical and structural factors on biomass recalcitrance and of the most advanced techniques to evaluate these factors. Also, recent spectral and water-related measurements accurately predicting hydrolysis are presented. Overall, combination of relevant factors and specific measurements gathering simultaneously structural and chemical information should help to develop robust and efficient LB conversion processes into bioproducts.

## Introduction

The environment is suffering from climate change, worsened by over-exploitation of resources thus increasing global greenhouse gas emission (Anderson et al., 2019; Hassan et al., 2019). Sustainable and environmentally friendly energy based on renewable resources are required in order to meet the world's future energy needs. Lignocellulosic biomass (LB) continues to attract global interest as a sustainable alternative to fossil carbon resources to produce second-generation biofuels and other biobased chemicals without compromising global food security (Menon and Rao, 2012; Chandel et al., 2018). These include agricultural wastes such as cereal straw (Yuan et al., 2018) and bagasse (Dias et al., 2009), forest residues such as pine (Cotana et al., 2014) and dedicated crops and short rotation coppices such as miscanthus (Lewandowski et al., 2000), switchgrass (Schmer et al., 2008), and poplar (Sannigrahi et al., 2010). LB is mainly composed of cellulose, hemicelluloses and lignin, making a complex assembly of polymers naturally recalcitrant to enzymatic conversion. That is why some pre-treatment steps are mandatory to make cellulose more accessible by changing the physical and/or the chemical structure of LB and facilitating the conversion of polysaccharides into fermentable sugars (Zhao et al., 2012a; Kumar and Sharma, 2017). Factors affecting LB recalcitrance are strongly interconnected and difficult to dissociate (Zhao et al., 2012b; Bichot et al., 2018). They can be divided into structural factors, which mainly refer to cellulose specific surface area, cellulose crystallinity, degree of polymerization, pore size and volume; chemical factors, related to composition and content in lignin, hemicelluloses and acetyl groups. Although many studies have investigated the impact of these factors on recalcitrance by examining different LB feedstocks and operating process conditions, conclusions obtained are not always obvious and even sometimes contradictory.

This review aims to propose an up-to-date survey of the role of chemical and structural factors on biomass recalcitrance and of the most advanced techniques to evaluate these factors. Resulting from the assessment of these factors, some promising methods aimed at predicting hydrolysis are presented and discussed, so that they should help to develop robust LB conversion processes into biofuels and biobased chemicals.

## Factors Contributing to the Recalcitrance of Lignocellulosic Biomass

LB is naturally recalcitrant to microbial and enzymatic degradation, which constitutes a real obstacle to its industrial valorisation into bioenergy and biomaterials. To optimize deconstruction, it is necessary to understand and overcome the chemical and the structural factors conferring the recalcitrance property to lignocellulose in plant cell walls.

### Chemical Factors Impacting Enzymatic Hydrolysis

#### Cellulose

Cellulose, the most abundant LB polymer, representing 40–60% in weight (Sharma et al., 2019), consists of ß-D-glucopyranose units linked via ß-(1,4) glycosidic bonds, with cellobiose as the fundamental repeating unit. The cellulose chains made up of 500–1400 D-glucose units are arranged together to form microfibrils, which are packed together to form cellulose fibrils (McKendry, 2002; Robak and Balcerek, 2018). Cellulose fibrils are embedded in a lignocellulosic matrix that makes it very resistant to enzymatic hydrolysis. Yoo et al. reported that the cellulose content was positively correlated with the glucose release (Yoo et al., 2017a). The degree of polymerization (DP) of cellulose which is the number of glucose units in the polymer playing a crucial role on LB recalcitrance. But its exact role is still not quite clear and difficult to investigate individually with the current knowledge. Indeed, altering DP is always accompanied by changes in structural parameters such as crystallinity and porosity. For example, Sinitsyn et al. (1991) found that reduction in DP of cotton linters by γ-irradiation had a minor effect on the saccharification rate. Ioelovich et al. got similar conclusion (Ioelovich and Morag, 2011). However, Lu et al. (2019) reported that the cellulose DP was negatively correlated to the cellulose hydrolysis. It is assumed that long cellulose chains contain more hydrogen bonds and are difficult to hydrolyze, whereas shorter cellulose chains contain a weaker hydrogen-bonding system and therefore are believed to facilitate enzyme accessibility (Hallac and Ragauskas, 2011; Meng et al., 2017).

#### Hemicelluloses and Acetyl Groups

Hemicelluloses are heterogeneous groups of biopolymers, representing 20–35% of the biomass weight (Chandel et al., 2018). It contains various monosaccharide subunits to form xylans, xyloglucan, mannans and glucomannans, and others (McKendry, 2002). The DP of hemicelluloses is in the range of 100–200 units (Mota et al., 2018), which is much lower than that of cellulose, but it can present a high degree of more or less complex substitutions. Hemicellulose is amorphous, with little physical strength. It is readily hydrolysed by dilute acids or bases, as well as hemicellulase enzymes (Isikgor and Becer, 2015). Hemicelluloses act as a physical barrier limiting the accessibility of enzymes. It has been reported that removal of hemicelluloses by dilute acid or steam explosion pre-treatment could increase cellulose conversion by improving the accessibility of enzymes to cellulose (Auxenfans et al., 2017a; Herbaut et al., 2018; Santos et al., 2018). Kruyeniski et al. (2019) reported that the removal of hemicelluloses on pre-treated pine improved the fibers porosity and the area available for enzymes. The impact of hemicelluloses on LB recalcitrance still not quite clear as some lignin is often removed with hemicelluloses. Some studies have reported that hemicelluloses removal was more efficient than lignin removal for improving enzymatic hydrolysis rate (Yoshida et al., 2008; Leu and Zhu, 2013; Lv et al., 2013), whereas others indicated that lignin removal was much more important (Gao et al., 2013; Kruyeniski et al., 2019).

LB hemicelluloses can be extensively acetylated with acetyl groups (OAc). OAc may restrict cellulose accessibility by interfering with enzyme recognition (Pan et al., 2006). It also might hinder the formation of productive binding between cellulose and the catalytic domain of cellulases through increasing the diameter of cellulose chain or changing its hydrophobicity (Zhao et al., 2012a). Previous studies on corn stover reported that reducing the acetyl content improved enzyme effectiveness (Kumar and Wyman, 2009a,b). Whereas, other studies on poplar wood, wheat straw, switchgrass and bagasse pointed out that the effect of deacetylation was more significant on hemicellulose digestibility than on cellulose digestibility (Grohmann et al., 1989; Chang and Holtzapple, 2000; Liu et al., 2014). Chang and Holtzapple (2000) and Zhu et al. (2008) showed that the impact of OAc depends on the lignin and cellulose content and biomass crystallinity.

#### Lignin

Lignin is the second most abundant polymer in LB after cellulose, corresponding to 15–40% of dry weight (Ragauskas et al., 2014). It is a very complex amorphous heteropolymer of phenylpropanoid building units (*p*-coumaryl, coniferyl, and sinapyl alcohol) (Agbor et al., 2011). Lignin is responsible for hydrophobicity and structural rigidity. It binds hemicelluloses to cellulose in the cell wall. It is well-known that lignin plays a negative role in the conversion of cellulose influenced by several factors such as total lignin content, lignin composition/structure (in particular hydroxyl groups content and S and G units content) (Santos et al., 2012). First of all, lignin can physically limit polysaccharide accessibility: it plays a role as physical barrier that blocks the access of enzymes to cellulose. Also, it can irreversibly adsorb cellulases and other enzymes during enzymatic hydrolysis due to its hydrophobic structural features including hydrogen bonding, methoxy groups, and polyaromatic structures (Kumar and Wyman, 2009b; Zeng et al., 2014). Previous studies showed that the lignin content was negatively correlated with enzymatic digestibility in poplar (Meng et al., 2017; Yoo et al., 2017a), also in miscanthus, in wheat straw (Herbaut et al., 2018) and in transgenic rice (Huang et al., 2017). The removal of lignin generally disrupts the lignin-carbohydrates matrix, increases the porosity and reduces non-productive adsorption sites for enzymes (Pihlajaniemi et al., 2016; Kruyeniski et al., 2019). It has been reported that phenolic hydroxyl groups (lignin-derived compounds) cause reversible inhibition of cellulases (Yu et al., 2014; Yang and Pan, 2016; Yao et al., 2018). Blocking free phenolic hydroxyl groups by chemical reaction such as hydroxypropylation significantly reduced (by 65–91%) the inhibitory effect of lignin (Yang and Pan, 2016). Yoo et al. showed that lignin S/G ratio is important as an independent recalcitrance factor (Yoo et al., 2017a). However, the correlation between lignin S/G ratio and recalcitrance is still not obvious. For example, Herbaut et al. and Yu et al. showed a positive correlation between the S/G ratio and the hydrolysis yields for miscanthus and woody chips (Yu et al., 2014; Herbaut et al., 2018) because of the higher binding capacity of G (with branched structure) over S (with linear structure and low degree of polymerisation) to cellulase (Guo et al., 2014; Yoo et al., 2017b). By contrast, others found a negative correlation between S/G ratio and the enzymatic hydrolysis in woody chips (Papa et al., 2012), in pre-treated miscanthus (Xu et al., 2012; Li et al., 2014), in pre-treated wheat straw (Jiang et al., 2016) and in genetically engineering poplar (Escamez et al., 2017). On the other hand, previous studies showed that changes in S/G ratio of untreated LB did not influence the enzymatic hydrolysis: for untreated poplar with S/G ratio between 1.0 and 3.0 (Studer et al., 2011), for Arabidopsis stems containing G- and S-rich lignin (Li et al., 2010) and for transgenic alfalfa (Chen and Dixon, 2007). Overall, lignin contributes strongly to LB recalcitrance influenced by its chemical composition and its structure, limiting the accessibility of enzymes to cellulose.

#### Interactions Between Polymers

We detailed the impact of each polymer on LB recalcitrance above. There is a need to find out how interactions between them increase the recalcitrance of the cell walls to the enzymatic hydrolysis. Cellulose and hemicelluloses are intimately associated together through hydrogen bonds (Lee et al., 2014), meanwhile lignin are covalently linked to hemicelluloses to form lignin-carbohydrate complex (LCC) (Tarasov et al., 2018; Giummarella and Lawoko, 2019). There are five different types of lignin-carbohydrate bonds, phenyl glycosides (PG), benzyl ethers (BE), γ-esters esters (GE), ferulate/coumarate esters (FE/CE) and hemiacetal/acetal linkages that are linked to lignin at 4-OH and 4-O positions (Giummarella and Lawoko, 2019). It has been suggested that the interactions between the microfibers from cellulose and hemicelluloses, as well as the LCC linkage plays a significant role in wood structure and affects significantly its enzymatic hydrolysis by reducing the area of cellulose accessible for enzymes (Balan et al., 2009; Du et al., 2014). Until now, the study of LCC is still a controversial topic in lignocellulosic chemistry, due to difficulties in the characterization of heterogeneous biomass substrate in addition to the low concentration of LCC (Obst, 1982). That is why there is a need to develop efficient methods to enrich the LCC such as mild chemical methods or the use of pure enzymes in order to achieve a quantitative analysis of LCC using NMR (Giummarella and Lawoko, 2019).

### Physical Factors Impacting Enzymatic Hydrolysis

#### Crystallinity

Crystallinity has been identified as one of the most extensively studied supramolecular properties of cellulose. It represents the proportion of crystalline regions to amorphous regions. Crystalline cellulose fibers are closely related to each other by non-covalent hydrogen bonds that make their enzymatic hydrolysis 3–30 times lower than in amorphous zones (Zhao et al., 2012b). But the impact of crystallinity on hydrolysis differs. Some studies reported that crystallinity correlated negatively with enzymatic hydrolysis especially at the initial hydrolysis rate on pre-treated wheat straw (Pihlajaniemi et al., 2016), on pre-treated corn stover (Liu et al., 2014; Xu et al., 2019) and on hybrid polar, switchgrass, and bagasse (Chang and Holtzapple, 2000). Others showed that crystallinity was less critical in limiting hydrolysis than other physical features such as DP, pore volume, accessible surface area, and particle size (Mansfield et al., 1999; Ioelovich and Morag, 2011; Aldaeus et al., 2015; Auxenfans et al., 2017a; Meng et al., 2017; Zhang et al., 2018). Another piece of evidence is that in the majority of cases, pure cellulose is used as a substrate to correlate the crystallinity to the saccharification yield, which is not representative of the heterogeneous of LB substrate.

#### Particle Size

Particle size was identified as a key parameter affecting cellulose hydrolysis potential (Barakat et al., 2014; Vaidya et al., 2016). The reduction of particle size through milling, grinding, and extrusion could enhance the affinity between cellulose and enzymes, deconstruct lignocellulose compact structure and thus increase the rate of hydrolysis (Silva et al., 2012; Pang et al., 2018; Yu et al., 2019). Studies have demonstrated that mechanical deconstruction facilitates enzymatic hydrolysis of various feedstocks such as wood chips (Jiang et al., 2017), miscanthus and wheat straw (Kim et al., 2018) and corn stover (Yu et al., 2019). However, some researchers pointed out that there is a size threshold depending on the lignocellulosic feedstocks. Chang and Holtzapple (2000), observed that a particle size reduction below 400 μm has a negligible effect on the hydrolysis yield of poplar. Whereas, Silva et al. (2012) reported that the size threshold was 270 μm for wheat straw.

#### Accessible Surface Area (ASA)

Accessible surface area (ASA) of LB is a critical factor for enzymatic hydrolysis, highly related to porosity structure properties, such as specific surface area (SSA) and pore volume (Liu et al., 2014). Reduction in the particle size or increase in pore volume causes an increase of ASA. It has been shown that theenzymatic conversion of pre-treated pine wood is increased with ASA (Torr et al., 2016). Also Goshadrou et al. (2013) reported that ASA could enhance the fiber accessibility of aspen wood to the hydrolytic enzymes. However, ASA is difficult to estimate, SSA is often used to measure the real surface that is available to enzymes (Silvi Octavia et al., 2017). Moreover, just like crystallinity, it is impossible to consider only SSA (Karimi and Taherzadeh, 2016a). The smaller the particle is, the higher the SSA is (Silvi Octavia et al., 2017). Zhang et al. (2018) reported that the hydrothermal pre-treated corn stover increased the SSA by 2-fold, resulting in 138% enhancement of enzymatic digestibility. Lu et al. (2019) also found that ball milling increased the SAA of cellulose by a factor of two due to the reduction in particle size; these changes made cellulose more accessible and more reactive and resulted in higher glucose yield. However, Peciulyte et al. (2015) pointed out the absence of significant correlation between the yield of cellulose conversion of cellulosic substrates and the SSA.

#### Accessible Volume (Pore Size: Internal Surface Area)

Accessible volume of cellulose in LB is considered as an important factor influencing enzymatic deconstruction (Jeoh et al., 2007). According to their sizes or their shapes, pore volumes are more or less accessible to enzymes. The size of a cellulase is typically around 5.1 nm, and so, only the pores larger than 5.1 nm are supposed to be accessible to enzyme (Grethlein, 1985). Some authors found that there is a strong correlation between pore size of the biomass and the enzymatic conversion yield for dilute acid pre-treated poplar (Meng et al., 2013) and cellulosic substrates (Peciulyte et al., 2015). Herbaut et al. demonstrated that correlations with specific porosity ranges are biomass specific and pre-treatment dependent. For example, hydrolysis yield correlated strongly to pore size range 15–30 nm for wheat straw, whereas it correlated to pore size range 10–15 nm for poplar. For miscanthus, only pores below 10 nm correlated strongly with hydrolysis, which proved that there is no generic pore size allowing an enhancement of hydrolysis yield and that diffusion of enzymes within the plant cell wall is specific to each biomass species (Herbaut et al., 2018). Other reports found that there is no correlation between pore size and hydrolysis yield, for dilute acid pre-treated corn stover (Ishizawa et al., 2007), pre-treated pine (Kruyeniski et al., 2019), and dilute acid pre-treated and delignified sugarcane (Santos et al., 2018). Moreover, Stoffel et al. (2014) and Vaidya et al. (2016) also showed that the increase of pore volume when lignin contents does not exceed 15% has a negligible effect on enzymatic digestibility of pre-treated pine.

## Predicting Enzymatic Hydrolysis

**Figure 1:**
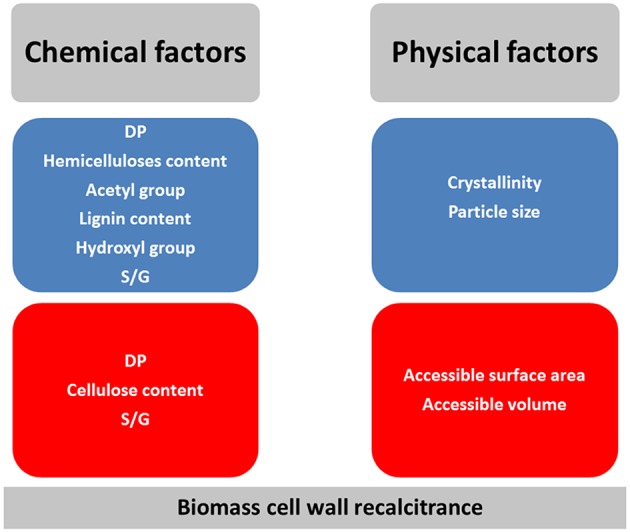
Factors influencing LB recalcitrance. In blue box: the increase in value of the factor increases LB recalcitrance; in red box: the increase in value of the factor decreases LB recalcitrance. When factors are in two boxes, their effect is variable.

LB recalcitrance to enzymatic degradation was found to be a multi-variant and multi-scale phenomenon, affected by several physical and chemical factors such as hemicelluloses and lignin content, DP of cellulose and accessible surface area and volume (Figure 1). However, various studies have demonstrated opposing trends in the effects of these factors due to the complexity of LB and the unknown interactions between these factors.

Several studies have developed high throughput methodologies to characterize the composition and the structure of large sets of LB samples using wet chemistry and spectroscopy (Studer et al., 2010; Krasznai et al., 2018). These methods have the advantages of being fast and automatic with a very low sample mass and minimal sample preparation (Decker et al., 2018). For instance, Selig et al. developed a high throughput method to determine glucan and xylan content in poplar, pine, wheat stover and pine using a glucose oxidase and a xylose dehydrogenase-based assays instead of HPLC analysis which reduced remarkably the duration of the analysis from 48 to 5-6 h (Selig et al., 2011). Pyrolysis-molecular beam mass spectrometry was used as a high throughput technique to analyse lignin content and structure and polysaccharides content in LB feedstocks (Penning et al., 2014; Decker et al., 2015; Sykes et al., 2015; Harman-Ware et al., 2017). Decker et al. (2012) developed a high throughput method to investigate the effect of the starch content in a set of 250 switchgrass variants on the recalcitrance after hydrothermal pre-treatment and enzymatic hydrolysis (5 days).

High throughput techniques provide useful information to characterize LB while saving time and effort. However, those techniques are not fully automatic (some manual transfers between operations is often necessary), also specialized costly robots, reactors, and sophisticated computational tools are required. More importantly, conducting enzymatic hydrolysis takes several days, whereas there is a need to predict from initial properties of biomass how it will behave during biotechnological transformation in order to adapt the process conditions.

As detailed in the previous section, measuring conventional factors such as lignin content (for example using standard wet-chemistry analysis), cellulose crystallinity or porosity (for example using Simons's staining or thermoporosimetry) provide reliable information about composition, accessibility and structure and can be carried out by several techniques with their advantages and drawbacks (Table 1).

**Table 1:** Approaches to study the chemical and physical factors influencing LB recalcitrance.

**Factors influencing LB recalcitrance**			**Techniques**	**Advantages**	**Disadvantages**	**References**
Chemical factors	Cellulose	Content	Acid-detergent methods Acid-hydrolysis followed by HPLC analysis	Quantitative analysis	Under estimation of cellulose content	Herbaut et al., 2018; Krasznai et al., 2018
		Degree of polymerization (DP)	Gel Permeation Chromatography (GPC)	Dedicated method	Dissolution of cellulose can be uncomplete	Engel et al., 2012; Meng et al., 2017
			Viscometry	No calibration	Instability of cellulose in the alkaline solvent	Karimi and Taherzadeh, 2016a; Neto et al., 2016
	Hemicelluloses	Content	Acid hydrolysis followed by HPLC analysis	Robust	Methyl esterified samples non detected	Herbaut et al., 2018
		Acetyl groups	Nuclear Magnetic Resonance (NMR)	Sensitive	Sample preparation	Arai et al., 2019
			Gas Chromatography-Mass Spectrometry (GC-MS)	Robust	Sample derivative preparation	Benouadah et al., 2019
			Acid-hydrolysis followed by HPLC analysis	Rapid	Global analysis	Li et al., 2015
	Lignin	Content	Acid hydrolysis followed by Klason lignin (insoluble) and UV absorbance (acid-soluble lignin).	Simple and relatively fast	Over estimation of the soluble lignin content	Auxenfans et al., 2017a
			Acetyl bromide soluble	Rapid, simple, precise, appropriate for small sample size	Lignin standard needed Sample solubilisation	Hatfield and Fukushima, 2005
		Molecular weight	GPC	Dedicated method	Formation of aggregates	Yoo et al., 2017a
		Phenolic hydroxyl group	NMR	Fast and sensitive	Sample solubilisation	Yao et al., 2018
			Infrared spectroscopy (IR) and Raman spectroscopy	Fast and sensitive	Only chemical functions analysis	
		Structural information	NMR	In-depth information, fast and sensitive	Complex sample preparation	Yao et al., 2018
			IR	Non-invasive, easy and fast	Only chemical functions analysis	Over et al., 2017
Physical factors	Crystallinity (CrI)		NMR	In-depth information and sensitive to the crystalline phase and amorphous phase	Results highly dependent on the instrument and the method used to analyse data	Yoo et al., 2017a
			X-Ray Diffraction (XRD)	High sensitivity to the crystalline phase	Qualitative estimation and lack of precision Depending on the method used to analyse data, the CrI value can vary drastically XRD is less sensitive to the amorphous phase	Lee et al., 2015; Chen et al., 2018
			IR and Raman spectroscopy	Semi-quantitative and sensitive Minimal sample preparation	Chemometric techniques required Only relative values	Monrroy et al., 2015; Agarwal et al., 2016
			Sum-Frequency-Generation (SFG) vibration spectroscopy	Selective detection of crystalline cellulose		Barnette et al., 2011; Lee et al., 2015
	Particle size		Laser granulometry	Fast and simple	Method based on the assumption that the particles are spherical, which is not always the case	Pang et al., 2018
			Scanning Electron Microscopy (SEM)	The morphology and microstructure of the particles can be observed and their sizes can be quantified	Image processing is needed	Vaidya et al., 2016; Pang et al., 2018
	Accessible surface area (ASA)		Simons' staining (SS)	Can be done in wet samples, measures both internal and external surface	Semi-quantitative Measurement depends on the shape and tortuosity of the pores	Meng et al., 2017; Santos et al., 2018
			Water Retention Value (WRV)	Reflects correctly the swelling of the lignocellulosic matrix	WRV depends on the chemical composition and the structure of biomass Overestimation of ASA	Weiss et al., 2017, 2018
	Pore volume		Mercury porosimetry	Provides a wide range of information: pore size distribution, total area and pore volume, average pore diameter	Suitable for macro-pores (14–200 μm) Over estimation of the pore volume (the smallest one)	Grigsby et al., 2013; Brewer et al., 2014; Meng et al., 2015
			Brunauer-Emmett-Teller (BET)	Sensitive	Over estimation of pore volume	Liu et al., 2019
			Solute exclusion	Quantitative	Does not determine accessibility of external surfaces	Ishizawa et al., 2007
			NMR cryoporometry and relaxometry NMR	Non-destructive	Requires complex setup	Meng and Ragauskas, 2014
			Thermoporometry (TP-DSC)	Simple	Over estimation of pore volume	Gustafsson et al., 2019; Kruyeniski et al., 2019
			Soft X-ray Tomography (SXT)	High resolution and quantitative information	SXT data collection is challenging	An et al., 2019
	Surface morphology		SEM and Transmission electron microscopy (TEM)	High resolution 2D images. Provide qualitative and quantitative informations.	Sample preparation may damage the samples	Karimi and Taherzadeh, 2016b; Li et al., 2018
			Atomic Force Microscopy (AFM)	No sample preparation	Low scanning speed	Isaac et al., 2018

In many cases, they are very useful to understand the relationship between assayed factors and hydrolysis. But these parameters are far from being universal to predict hydrolysis, for several reasons:

– Hydrolysis conditions depend on biomass species, pre-treatments, and enzymatic cocktails, which are not standard from one research report to another;
– Measurements of the chemical and structural parameters depend on the instrument, methods and conditions of analysis which are difficult to compare (for example, there are at least 6 different techniques to evaluate porosity, Table 1);
– Most importantly, due to the multi-scale architecture of LB, not a single chemical or structural parameter can so far explain hydrolysis.

Therefore, some other methods are required to evaluate, as a single measurement, the interactions existing between several parameters which are responsible for recalcitrance, in order to predict enzymatic hydrolysis. Among them, spectral and water-related properties of LB appear as relevant measurements to be correlated to hydrolysis.

### Infra-Red Spectroscopy

Quantitative spectroscopy is a fast, relatively low-cost, non-destructive alternative to classic analytical methods for the chemical analysis of biomass. Several spectroscopic techniques have been applied to analyse biomass properties, including fast-Fourier Transform InfraRed (FT-IR) spectroscopy, near-infrared (NIR) spectroscopy, and Raman scattering spectroscopy.

NIR is a good method for qualitative and quantitative screening of large population of samples. It has also been used to predict LB composition and enzymatic digestibility (Hou and Li, 2011; Huang et al., 2012, 2017).

FTIR spectroscopy is a reliable technique used to determine the effect of pre-treatment (monitor the crystallinity changes), and the degradation of cellulose during the enzymatic saccharification. However, it provides especially qualitative structural information rather than quantitative information. Bekiaris et al. (2015) demonstrated that FTIR spectroscopy combined with chemometric techniques can be used to predict sugar conversions and yields from enzymatic saccharification of pre-treated wheat straw. Earlier study on untreated and pre-treated switchgrass and corn stover got similar conclusion (Sills and Gossett, 2012). Raman spectroscopy is also a robust analytical technique coupled to FTIR spectroscopy allowed the prediction of lignin syringyl/guaiacyl content in diverse lignocellulosic feedstocks (Lupoi et al., 2014).

Overall, even if infrared spectroscopy is very powerful to predict the composition and the saccharification of LB, it requires the creation of mathematical models which needs hundreds of samples, which represents an intensive work. Also, models are specific to biomass species and hydrolysis conditions, which can limit their use.

### Fluorescence Spectroscopy

Plant cell walls are autofluorescent materials, containing some endogenous fluorophores, especially aromatic molecules: monolignols in lignin, ferulic, acid and cinnamic acids in hemicellulose (Auxenfans et al., 2017b). Fluorescence can be easily and fastly measured on lignocellulosic samples through spectrofluorimetry. Auxenfans et al. reported that fluorescence intensity correlated strongly with the glucose released from untreated and steam exploded lignocellulosic feedstocks (miscanthus, poplar, and wheat straw) and concluded that fluorescence can predict the LB saccharification.

Fluorescence lifetime as a rapid method can also be used to explain and even predict saccharification with efficiency: Chabbert et al. (2018) reported a strong positive correlation between lifetime fluorescence and saccharification yields. Fluorescence Recovery After Photobleaching (FRAP) technique allowed to explore LB accessibility. Herbaut et al., studied the mobility of PEG-rhodamine probes in cell walls of poplar samples and demonstrated a strong correlation between the accessibility of probes and the saccharification yields (Herbaut et al., 2018). Overall, even if fluorescence by itself cannot be related to a single parameter (lignin content, polymer interactions,…), this can be turned into an advantage since it provides a fingerprint of lignin organization and architecture in LB, which is likely directly related to cellulose accessibility and thus to hydrolysis potential.

### Water-Related Properties

Water acts as a swelling agent allowing enzymes diffusion toward plant cell wall. It has been reported that water retention value (WRV) can serve as predictor of hydrolysis rate (Noori and Karimi, 2016; Crowe et al., 2017; Williams et al., 2017; Paës et al., 2019). WRV is a complex function related to chemical and structural properties of the cell wall, such as accessible surface area and particle size. Several works reported a positive correlation between WRV and cellulose conversion rate for various lignocellulosic feedstocks: untreated maize (Li et al., 2015), pre-treated poplar, pine and miscanthus with dilute sulfuric acid (Weiss et al., 2018) and pre-treated corn stover and switchgrass with liquid hot water (Williams and Hodge, 2014). This method is rapid, simple, inexpensive, and provides results comparable to the results of more advanced methods, e.g., NMR and Simons' staining (Karimi and Taherzadeh, 2016a).

Another experimental parameter related to water that can be easily and fastly measured is the contact angle value that indirectly quantifies the hydrophobicity of lignin. This parameter could predict the inhibitory effect of lignin. Yang et al. reported that softwood lignin more hydrophobic than the hardwood lignin, was more able to absorb cellulase and inhibit enzymatic cellulose hydrolysis than the hardwood lignin (Yang and Pan, 2016).

## Summary and Perspectives

Biomass recalcitrance is a multi-variant and multi-scale phenomenon, and thus cannot simply be assayed by one single chemical or structural factor due to the complex and still unknown interactions between these parameters (Figure 1). Nonetheless, spectral analysis based on infrared and fluorescence properties of LB together with water-related characteristics seem to be able to represent chemical and structural properties of LB, thus relating nano- and macro-scale properties. Regarding future developments, the integration of large amount of data (chemical properties, images, spectra) by the means of machine learning approaches should help devising more complex models predicting not only composition but also dynamical behavior of LB over transformation such as hydrolysis. In this context, imaging and quantification of structural features at the cellular/tissular scale (by fluorescence confocal microscopy based on previous reports studying for example plant morphogenesis) or at nano-scale (by atomic force microscopy) might be relevant paths to follow.

## Author Contributions

AZ and GP discussed the outline, content of the article, and approved the content of the manuscript. AZ drafted the manuscript. GP finalized the manuscript.

### Conflict of Interest

The authors declare that the research was conducted in the absence of any commercial or financial relationships that could be construed as a potential conflict of interest.
